# Automated Health Care Thematic Analysis Using a Multiagent Large Language Model: Algorithm Development and Evaluation Study

**DOI:** 10.2196/90872

**Published:** 2026-09-30

**Authors:** Qidi Xu, Nuzha Amjad, Grace Giles, Alexa Cumming, De'angelo Hermesky, Alexander Wen, Min Ji Kwak, Yejin Kim

**Affiliations:** 1 The University of Texas Health Science Center at Houston Houston, TX United States

**Keywords:** human-AI collaboration, large language model, multiagent framework, qualitative research, thematic analysis

## Abstract

**Background:**

Understanding patients’ experiences is essential for advancing patient-centered care, especially in chronic diseases that require ongoing communication. Qualitative thematic analysis is widely used to explore these experiences; however, the process remains labor-intensive, subjective, and difficult to scale.

**Objective:**

This study aimed to develop and evaluate Collaborative Theme Identification Agents (CoTI), a multiagent large language model framework designed to support manual thematic analysis by rapidly generating supporting excerpts, initial codes, and themes.

**Methods:**

CoTI consists of 3 agents: Instructor, Thematizer, and CodebookGenerator. The Instructor refines instruction prompts, the Thematizer extracts supporting excerpts and generates initial codes for each transcript, and the CodebookGenerator groups similar codes across all transcripts into a codebook with themes. We evaluated CoTI primarily using 12 transcripts of patient with heart failure, with a focus on perceptions of medication intensity. CoTI-generated outputs were compared against the reference standard developed by senior investigators. To explore human-AI interaction in thematic analyses, we further implemented CoTI in a user-facing application.

**Results:**

CoTI generated supporting excerpts, initial codes, and themes that were more similar to those of senior investigators than were the outputs of junior investigators, baseline natural language processing models, and other basic large language models. In an exploratory human-AI collaboration experiment, we found that the collaboration between CoTI and junior investigators provided only marginal gains compared to CoTI alone. A possible hypothesis was that junior investigators may overrely on CoTI and limit their independent critical thinking.

**Conclusions:**

CoTI can improve the efficiency of thematic analysis by rapidly generating supporting excerpts, initial codes, and themes for human researchers’ review. These findings highlight CoTI’s potential as a useful tool for scalable qualitative research.

## Introduction

Patient-centered care prioritizes understanding and integrating patients’ individual needs, values, and preferences into clinical decision-making [1]. This approach is particularly important in the management of chronic diseases such as diabetes, hypertension, and heart failure, which require long-term treatment plans and frequent communication between patients and health care providers to ensure timely adjustments in response to changes in the patient’s condition [2,3]. To explore how patients perceive and navigate their health experiences, qualitative research, particularly through thematic analysis of interview transcripts, has been widely used, offering rich sociocontextual understanding of complex health phenomena [4,5]. For example, previous qualitative studies in heart failure have used thematic analysis to identify themes related to medication intensity and self-management capacity [6,7].

Although thematic analysis has been widely used to generate valuable insights in patient-centered care, it also faces several practical challenges. As a traditional qualitative approach, it typically relies on trained experts to manually review transcripts, extract supporting excerpts (referred to as *clues*), interpret their meanings to identify initial codes, and summarize these codes into a codebook with themes for interpretation (Figure 1A). This process is time-consuming, labor-intensive, and susceptible to subjective interpretation, as experts may differ in how they extract and summarize information. These limitations may introduce variability and potential bias into the findings [8]. To address the challenges of manual thematic analysis, researchers have explored the use of natural language processing (NLP) to assist with thematic analysis [8]. Traditional unsupervised NLP techniques, such as latent Dirichlet allocation (LDA) [9], identify recurring patterns in word cooccurrence to generate clusters of keywords. These keyword lists are then interpreted by human analysts to assign themes. For example, Abram et al [10] used LDA to identify themes for nurse interviews in the substance use field. However, this still required manual review of model-generated keywords to identify meaningful themes. Supervised NLP techniques, such as supervised BERTopic [11], aim to identify themes that align with human predefined labels. However, thematic analysis is fundamentally an inductive process, where researchers typically begin with a small number (10-20) of transcripts to discover previously unknown themes. Because this task is to generate themes rather than apply existing ones, supervised NLP approaches are conceptually incompatible with thematic analysis. As a result, traditional NLP approaches either depend on human interpretation or struggle to adapt supervised frameworks to inductive discovery, making them less adaptable to the dynamic, context-rich narratives characteristic of qualitative health care research.

Recent advances in large language models (LLMs), such as GPT-4 [12], offer promising solutions to these limitations. LLMs can analyze long text and generate human-readable outputs in zero-shot or few-shot settings, only requiring instructions or a small number of labeled examples. This capability makes LLMs particularly well-suited for qualitative research contexts that lack annotated data or heavily rely on manual interpretation, effectively addressing key limitations of traditional NLP approaches. Prior studies have demonstrated the potential of LLMs in qualitative analysis. For example, Renard et al [13] suggested that LLMs can uncover hidden insights from patient interview transcripts and identify dominant themes. Similarly, Mannstadt et al [14] demonstrated that LLMs can rapidly identify dominant themes from patient interview transcripts, serving as a helpful complement to human analysis. Another study applied LLMs to identify themes about cancer patients’ experiences, demonstrating that LLMs perform well in capturing structural, temporal, and logistical aspects of narratives [15]. However, because these instructions were broad, the outputs often defaulted to generic patterns and overlooked emotional nuance and contextual depth.

To address these gaps, we developed Collaborative Theme Identification Agents (CoTI) to support qualitative thematic analysis with LLMs. Although frameworks such as Thematic-LM [16], Thematic Analysis Framework Using Multiagent LLM (TAMA) [17], and Auto-TA [18] have pioneered the use of multiagent systems for social media and clinical interview data, our framework is specifically designed to capture the objective-specific insights often overlooked by general-purpose agents. CoTI integrates 3 specialized agents: Instructor is responsible for producing tailored instruction prompts to capture objective-specific insights, including psychosocial, emotional, and contextual dimensions that are often overlooked by broad instructions, while Thematizer and CodebookGenerator are designed to reflect the 2 key phases of the analytical workflow, with Thematizer extracting supporting excerpts (clues) and identifying initial codes for each transcript and CodebookGenerator summarizing these codes with similar meanings across all transcripts into themes, as each transcript contained its own set of codes that often overlapped conceptually but varied in wording. While CoTI is capable of operating as a fully automated system, LLMs are often used alongside human researchers in practice, tasked with reviewing, refining, or validating LLM-generated outputs. However, how human-AI collaboration can enhance thematic analysis quality remains insufficiently understood. To explore this, we embedded CoTI’s Thematizer in a user-facing application that enables real-time interaction with humans. This implementation provides an opportunity to examine whether combining LLMs with human involvement can improve the quality of thematic analysis in health care research contexts.

Overall, the aim of this study was to develop and evaluate a multiagent LLM framework for improving the efficiency of qualitative thematic analysis and to examine whether human-AI collaboration can enhance the quality of thematic analysis in health care research.

**Figure 1:**
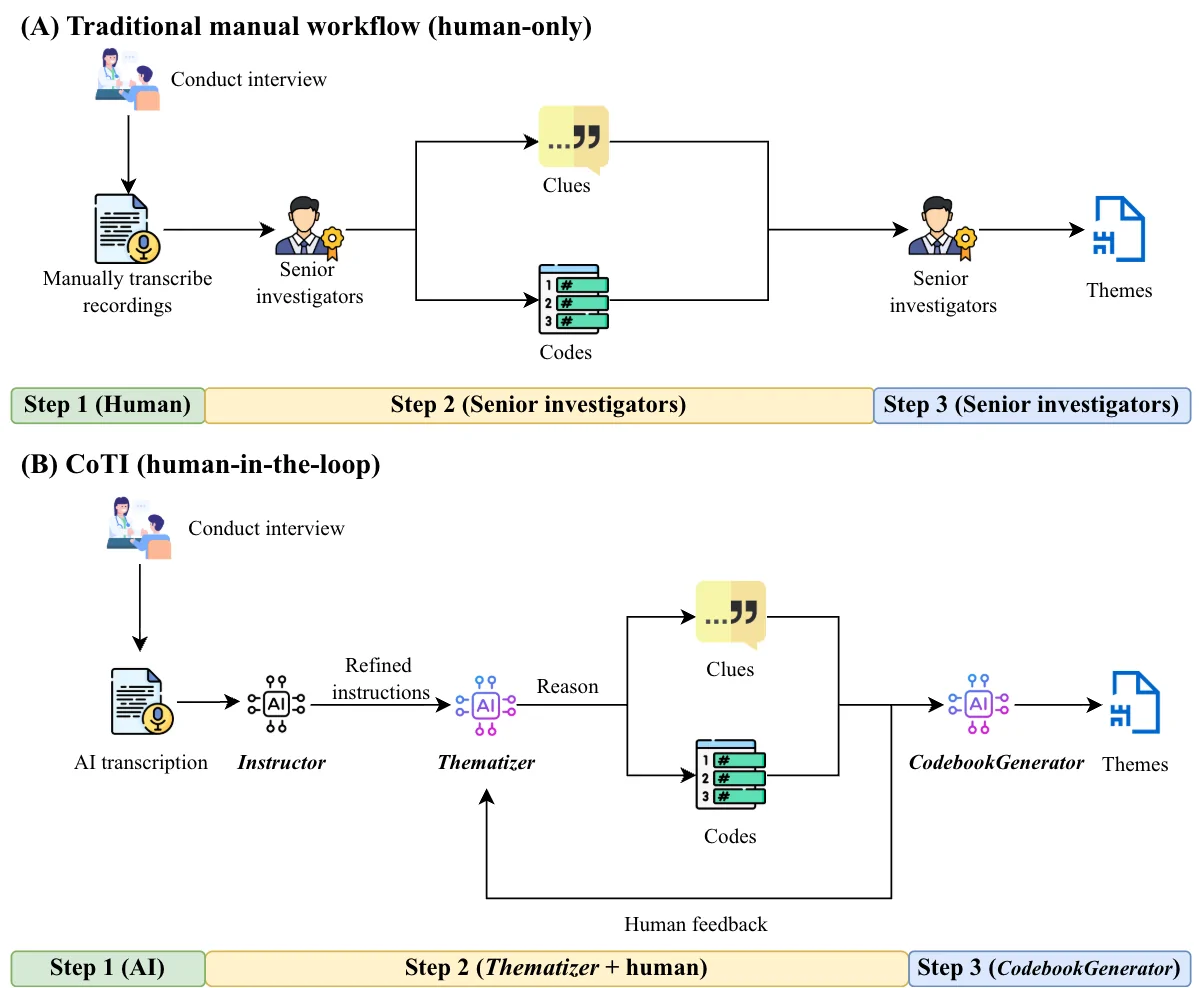
Study overview. To explore treatment burden perceptions among older adults with heart failure, our study compared the traditional thematic analysis with our proposed Collaborative Theme Identification Agents (CoTI) framework. (A) Traditional workflow: The manual method consists of 3 main steps. First, researchers conduct semistructured interviews and manually transcribe the audio recordings. Next, several senior investigators review each transcript to extract clues and identify initial codes. Finally, senior investigators generate themes based on clues and codes across all transcripts. This method is highly time-consuming and heavily reliant on expert labor. (B) CoTI workflow: the CoTI framework introduced a human-AI collaborative workflow that improved scalability while preserving human involvement. First, interviews were transcribed using Whisper, an automatic speech recognition tool. Before thematic analysis, Instructor (based on a heavyweight reasoning-oriented large language model [LLM]) iteratively improved the instruction prompts that guided thematic analysis. Once finalized, these instructions were injected into Thematizer (based on a lightweight general-purpose LLM) to extract clues, generate reasoning, and identify initial codes. Human review (eg, by junior investigators) and feedback on these AI-generated outputs is used to refine them. Outputs across all transcripts are processed through CodebookGenerator (based on a lightweight general-purpose LLM) to generate themes.

## Methods

### Data Collection

We used 10 interview transcripts that our research team collected for our previous study [6] and conducted 2 more interviews for this study. Specifically, we conducted a qualitative study using one-on-one, semistructured interviews with older adults (age ≥65 years) who were hospitalized in the acute cardiac care units at Memorial Hermann Hospital at Texas Medical Center and excluded patients diagnosed with heart failure for the first time during the hospitalization, those unable to respond appropriately due to mental status changes, or those who declined to participate [6].

The interview guide focused on four key questions: (1) participants’ perceptions of their heart medication intensity, (2) situations in which they would feel the medications excessive, (3) factors that would make medication management easier, and (4) their overall issues in medication management [6]. After conducting in-person interviews with each participant in the hospital, we transcribed the audio recordings via both professional transcription and OpenAI’s Whisper model (small size) [19]. Transcripts were reviewed by interviewers to ensure accuracy.

### Thematic Analysis Setting

We used 4 different settings to perform thematic analysis. First, 2 senior investigators independently extracted clues and identified initial codes for each transcript and developed the codebook with themes using inductive and deductive thematic analysis. Two additional independent senior investigators then reviewed all transcripts and refined themes [6]. These final themes served as the reference standard in our study. Second, 4 junior investigators (working independently from senior investigators) were assigned a subset of transcripts (Table S3 in Multimedia Appendix 1) and independently reviewed the transcripts to identify initial codes manually. Third, our proposed CoTI framework was applied to automatically extract clues and identify initial codes for each transcript and grouped similar codes across all transcripts to generate themes. Fourth, the same junior investigators used the web-based version of CoTI (see the Human-AI Collaboration Web-Based Application section) to extract clues and identify initial codes for their assigned transcripts.

### CoTI Model

#### Model Summary

CoTI implements its multiagent design through 3 LLM agents to mimic the traditional thematic analysis phases proposed by Braun and Clarke [20] in 2006 (Figure 1). Specifically, CoTI operationalizes phases that are relatively structured and reproducible, including familiarization, coding, and theme review [20]. We adopted a multiagent design because these steps involve distinct analytical functions. Separating these functions improves transparency and allows intermediate outputs to be reviewed more easily than a single LLM. Within this design, we implemented Instructor using the QwQ-32B (Alibaba Cloud) reasoning model [21] to generate high-quality, tailored instruction prompts that guide Thematizer’s analysis toward capturing contextual depth, thereby supporting the familiarization phase. Thematizer was built on the GPT-4o-mini (OpenAI) model [22] and extracts clues, generates reasoning, and identifies initial codes for each transcript, thereby reproducing the coding phase of the traditional thematic analysis while also allowing fast and efficient collaboration with humans to refine its outputs. CodebookGenerator, also based on the GPT-4o-mini model, summarizes similar codes across all transcripts into themes, supporting the theme reviewing phase.

This study used the GPT-4o-mini model, released on July 18, 2024 [22], and the QwQ-32B model, released on March 5, 2025 [21]. Our research team previously published a paper about patients’ perceptions of heart failure medications on February 4, 2025 [6]. Although there was a slight temporal overlap between the release of QwQ-32B and our earlier publication, we believe that all model development and data analyses had been completed prior to the release of QwQ-32B. Therefore, there was no possibility of data leakage or model memory.

#### Instruction Generation Phase

##### Overview

In order to obtain high-quality instruction prompts for guiding thematic analysis while minimizing expert labor, we implemented an iterative instruction refinement process using Instructor. It began with the random selection of several transcripts, which were submitted to a reasoning model to identify initial codes. These AI-generated codes were not treated as the final codes for the transcripts but served as provisional references to guide instruction prompt development. Instructor took these AI-generated codes as inputs and progressively produced the refined clue and reasoning instruction prompts through 4 interconnected stages: clue instruction, reasoning instruction, evaluation, and optimization. Each stage built upon the previous one, ensuring a systematic progression toward high-quality clue and reasoning instruction prompts (Figure 2). Importantly, no senior investigator-derived reference standard was provided during this process. The optimization procedure relied solely on the study objective, transcript content, and the provisional AI-generated codes.

**Figure 2:**
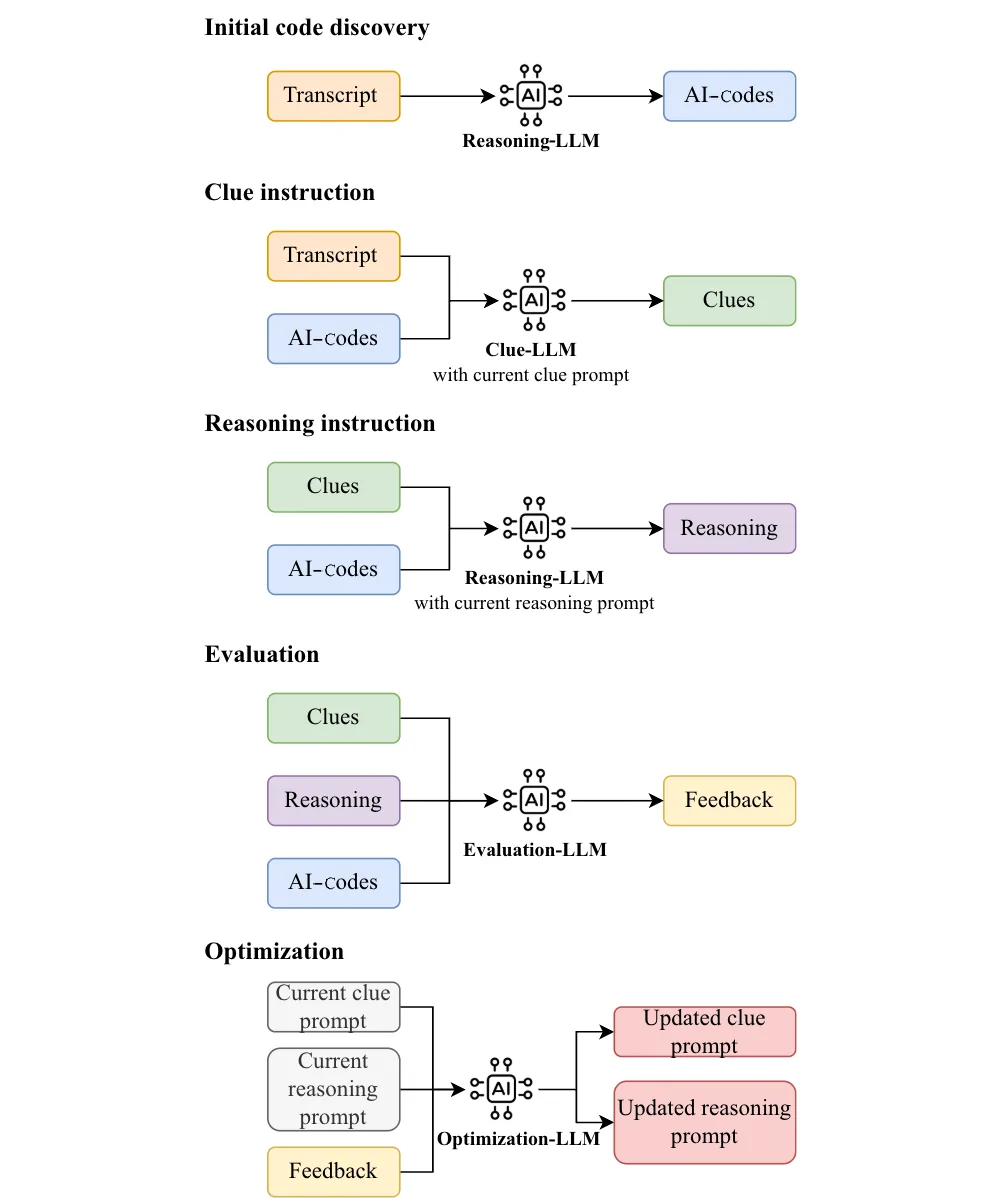
Workflow of instructor. Instructor is a multiagent framework designed to iteratively refine instruction prompts for Thematizer to perform thematic analysis. The process begins with a reasoning model (QwQ-32B) generating initial codes from several randomly selected transcripts. Instructor then launches a 4-agent optimization cycle: clue-large language model (LLM), reasoning-LLM, evaluation-LLM, and optimization-LLM. First, clue-LLM, using the current clue prompt, takes each transcript and its corresponding AI-generated codes as input to generate clues for each code. Then, reasoning-LLM, guided by the current reasoning prompt, receives these clues and codes to generate reasoning statements. Next, the evaluation-LLM takes every (clue, reasoning, and code) triplet to produce feedback outlining issues with the current prompts and suggestions for improvement. Finally, the optimization-LLM takes this feedback with the current clue and reasoning prompts to generate updated versions. These updated prompts replace the previous ones, initiating the next iteration of refinement.

##### Initial Code Discovery

To initiate the refinement process, we used the QwQ-32B reasoning model to identify initial codes in 2 randomly selected transcripts. The model was prompted to identify codes related to patients’ perceptions of the treatment burden or intensity of heart failure medications (see prompts in Textbox S1 in Multimedia Appendix 1).

##### Clue Instruction

The first stage of refinement focused on extracting clues to support the interpretation of identified codes. For each training transcript paired with its corresponding AI-generated codes, we prompted an LLM agent (referred to as clue-LLM, implemented using the QwQ-32B reasoning model) with an initial instruction, “list clues (ie, key phrases, contextual information, semantic and emotional tones, temporal information, symptom descriptions) in the following patient-doctor dialogue that support each given identified topic.” [23] (see prompts in Textbox S2 in Multimedia Appendix 1). This instruction guided the model to find supporting excerpts for each code directly from the transcript.

Clue-LLM was specifically instructed to find clues in the form of direct quotes from transcripts, as these preserve the exact language and context, ensuring the original meaning. Unlike summaries or interpretations, direct quotes provide original and unchanged references, reducing bias and enhancing transparency. They also serve as reliable and traceable memory units, locating specific parts of the interview.

##### Reasoning Instruction

The second stage aimed to formalize the logical relationships between the extracted clues and their associated codes through structured reasoning. Using the clues generated by clue-LLM, we prompted a second LLM agent (referred to as reasoning-LLM, implemented using the QwQ-32B reasoning model) with an initial instruction: “Based on the given clues, generate the reasoning process that supports the identified topics.” [23] (see prompts in Textbox S3 in Multimedia Appendix 1). This enabled the model to generate structured reasoning statements that clarified how the provided clues supported each code, thereby enhancing the clarity and explainability.

##### Evaluation

The third stage involved assessing the quality of the generated clues and reasoning. Rather than collecting feedback for each individual (clue, reasoning, and code) pair, we prompted a third LLM agent (referred to as evaluation-LLM, implemented using the QwQ-32B reasoning model) to identify common issues and offer overall suggestions for improving clue and reasoning instructions (see prompts in Textbox S4 in Multimedia Appendix 1). This aggregated feedback addressed several limitations associated with individual feedback. Individual evaluations may result in inconsistent insights and overemphasize isolated patterns while overlooking systemic issues. Additionally, providing detailed feedback for each pair may increase complexity and reduce clarity in the evaluation process. By synthesizing feedback across multiple training examples, evaluation-LLM was able to provide a more comprehensive assessment, enabling consistent and scalable improvements.

##### Optimization

The final stage focused on refining the clue and reasoning instructions based on feedback provided by evaluation-LLM. During this step, a fourth LLM agent (referred to as optimization-LLM) simultaneously improved the clue and reasoning instructions to address previously identified issues and enhance their overall quality. To mitigate the impact of potentially spurious feedback from the evaluation-LLM, we prompted optimization-LLM (implemented using the QwQ-32B reasoning model) with the instruction “The feedback may be noisy, identify what is important and what is correct.” [24] (see prompts in Textbox S5 in Multimedia Appendix 1). This prompt encouraged optimization-LLM to apply critical thinking, allowing it to prioritize relevant and accurate suggestions. The overall refinement process in the preprocessing phase was iterative, involving multiple cycles of clue and reasoning generation, evaluation, and optimization.

#### Thematic Analysis Phase

##### Overview

The thematic analysis phase aimed to apply the refined clue and reasoning instruction prompts to identify initial codes for all transcripts using Thematizer and subsequently summarize these codes across all transcripts into a codebook with themes by CodebookGenerator.

##### Codes Identification

To enable rapid code identification for a given transcript, particularly in the setting where junior investigators’ feedback is incorporated, we prompted Thematizer (implemented using the GPT-4o-mini model, which has faster inference speed compared with the QwQ-32B model used in Instructor) with the refined clue and reasoning instructions obtained from the instruction generation phase (see prompts in Textbox S6 in Multimedia Appendix 1). To capture a broader set of candidate codes, we repeated the code identification process 3 times and took the union of all codes generated across the 3 runs (see prompts in Textbox S7 in Multimedia Appendix 1).

##### Themes Generation

Because each transcript had its own set of codes after Thematizer, many of which overlapped but differed in wording, we used CodebookGenerator (implemented using the GPT-4o-mini model, for its faster inference speed) to group similar, semantically related, or duplicate codes across all transcripts into higher-level themes (see prompts in Textbox S8 in Multimedia Appendix 1). Each resulting theme included a theme name, a description, the original codes included, and representative clues, which was suitable for human review.

##### Human-AI Collaboration Web-Based Application

We converted the CoTI framework into a user-friendly, web-based application to facilitate interaction between human (eg, junior investigators) and our model (Figure 3). The application was built on Thematizer, which is responsible for extracting clues and identifying initial codes from individual transcripts. Because themes were generated through the analysis of all transcripts, and junior investigators in our study were each assigned only a subset of transcripts, CodebookGenerator was not converted into a human-collaboration module in our design. We selected a COVID-19 transcript as a demonstration example [25]. Within the application, users (eg, junior investigators) are prompted to enter their Azure API credentials and upload a transcript. The uploaded transcript is displayed in a viewing panel on the right-hand side of the interface. Upon clicking the “Process Transcript” button, Thematizer automatically analyzes the transcript to extract clues and identify corresponding initial codes. The outputs are displayed for user review, allowing them to locate the relevant text segments in the transcript using keywords derived from the extracted clues (Figure 3A). If users are not satisfied with the outputs, they are offered 2 options: “Try Again,” which reprocesses the transcript without feedback, or “Provide Feedback,” which refines the model’s outputs based on the user’s input. This iterative feedback loop continues until the user indicates satisfaction by selecting “I’m Satisfied,” at which point the final results are saved (Figure 3B).

**Figure 3:**
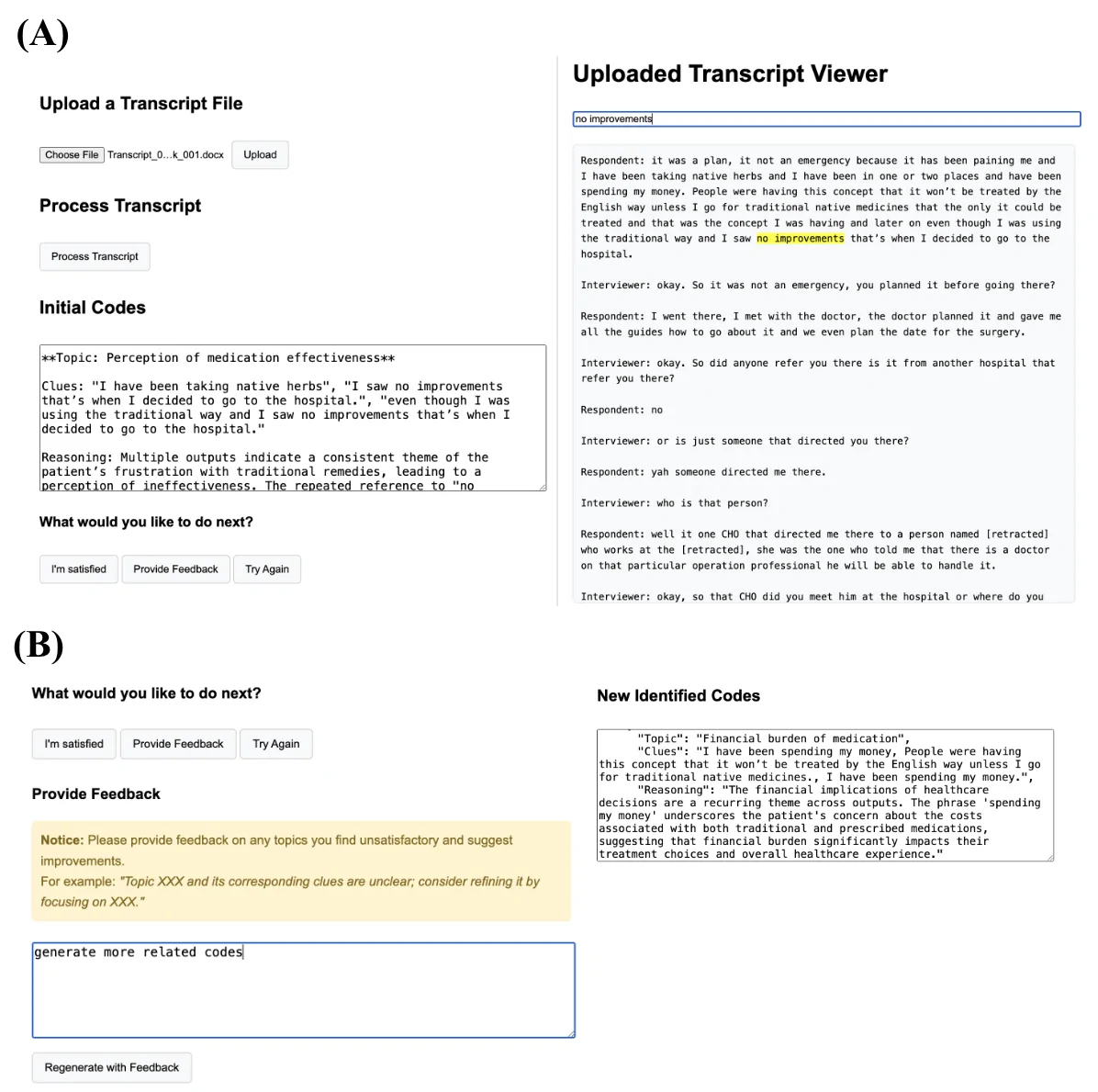
Web-based application. (A) After entering the Azure OpenAI API credentials and uploading a transcript, users can view the content on the right side of the interface. (B) Once the initial model-generated response is provided, users are presented with 3 options: “I’m satisfied,” “Try again,” and “Provide feedback.” Selecting “I’m satisfied” indicates that the user accepts the response and concludes the code identification process. Choosing “Try again” prompts the model to reprocess the transcript and generate a new response. If “Provide feedback” is selected, the application displays text boxes where users can enter feedback. The model then incorporates this feedback to refine its previous response.

### Evaluation Against Senior Investigators

Because qualitative analysis is inherently interpretive rather than purely objective, thematic analysis cannot be considered a simple pattern-recognition task. Accordingly, the purpose of our evaluation was not to determine whether model outputs were objectively correct, but to assess how completely the model captures insights identified by senior investigators.

To achieve this, we evaluated our model’s output (clues, codes, and themes) against those provided by senior investigators, which served as the reference standard. We first compared model-extracted clues with those provided by senior investigators for each transcript. To quantify similarity, we used multiple metrics, including Jaccard similarity, precision, recall, and *F*_1_-score. Jaccard similarity measured the degree of overlap between model-extracted and senior-extracted clues. Precision reflected the proportion of correctly extracted clues among all extracted clues, while recall captured the proportion of reference clues that the model successfully extracted. Next, to evaluate code similarity, we calculated the cosine similarity between model-identified codes and those identified by senior investigators using embeddings generated from OpenAI’s text-embedding-3-small model, which was independent of the models used in our main framework. Specifically, for each reference code, we identified the maximum cosine similarity with any model-generated code, averaged these values across all reference codes, and then averaged them across all transcripts. Finally, we assessed theme similarity by comparing the model-generated themes with the themes generated by senior investigators, using the same embedding-based cosine similarity method applied in the code evaluation.

### User Perception Survey

To understand junior investigators’ perceptions of CoTI’s outputs and their experience, we implemented a survey guided by the quality, understanding, expression, safety, and trust (QUEST) framework [26] (see detailed questionnaire in Section B in Multimedia Appendix 1). We had 4 independent junior investigators (medical school students who were not involved in the original theme generation project conducted by senior investigators) participating in this phase. Each junior investigator first reviewed their assigned transcripts to identify relevant codes manually and then used the web-based version of the CoTI application to perform the same task, after which they completed the survey.

### Baseline Models

We established 2 types of baseline models for comparison. The first type included traditional unsupervised topic modeling methods such as LDA [9], Top2Vec [27], and BERTopic [28]. These methods were selected because they are commonly used NLP approaches for identifying latent topics in textual data. However, they are not designed to perform the full qualitative thematic analysis workflow, such as extracting supporting excerpts and identifying initial codes for each transcript. Particularly, these baseline models take all transcripts as input and typically require preprocessing steps including data cleaning, tokenization, stopword removal, and lemmatization. Each baseline model outputs a set of latent themes represented as ranked lists of high-probability keywords. Since these keyword-based representations differ from human-interpretable themes, we developed a standardized evaluation framework for comparison. Specifically, we extracted the top 6 keywords from each baseline model’s output as proxies for the generated themes. We then converted both keywords and senior investigators-generated themes into embeddings and computed cosine similarity between them to assess theme quality.

The second baseline type (referred to as basic LLM) used a reasoning-oriented model (QwQ-32B) without refined instructions for clue extraction and reasoning generation. Because reasoning-oriented LLMs typically have longer inference times (GPT-4o-mini model required approximately 1 minute per transcript to complete 3 runs with aggregation, whereas a single run of QwQ-32B took approximately 3 minutes per transcript), we evaluated this baseline using a single run per transcript rather than the multiple aggregated runs used for CoTI. Unlike traditional models, this baseline was capable of generating clues, codes, and themes, allowing for direct comparison against senior investigators across all components.

### Ethical Considerations

The interview study was conducted in accordance with the Declaration of Helsinki and approved by the institutional review board (IRB) of the University of Texas Health Science Center at Houston (HSC-MS-21-0874). This study was approved by the Committee for the Protection of Human Subjects of the University of Texas Health Science Center at Houston (protocol HSC-SBMI-13-0549).

## Results

### Overview

We developed CoTI, a multiagent human-AI collaborative framework designed to automate qualitative thematic analysis. Our goal was to efficiently generate high-quality outputs (clues, codes, and themes) that were similar to those produced by human experts (eg, senior investigators). We evaluated CoTI’s performance across three tasks: (1) clue extraction (an intermediate output, ie, supporting excerpts from transcripts), (2) code identification (the primary output, ie, codes derived from clues) within each transcript, and (3) theme generation (across all transcripts, summarizing all codes into a codebook with themes). Our experiments showed that clues, codes, and themes that were identified by CoTI were more similar to those of senior investigators than were the outputs of traditional NLP models, basic LLMs, or human researchers with lower levels of experience (eg, junior investigators). Moreover, collaboration between CoTI and junior investigators did not lead to outputs that were more similar to those of the senior investigator than CoTI alone.

### Interview Data Collection and Patient Characteristics

As a case study, we conducted interviews with 12 patients with heart failure from Memorial Hermann Hospital at the Texas Medical Center in Houston, Texas, to explore their perceptions of challenges in using heart failure medications [6]. Among the 12 participants, 8 (66.67%) were female, with a mean age of 74.75 (SD 8.21) years. 5 (41.67%) participants were White, and 5 (41.67%) were African American. Detailed demographic and clinical information for each participant is presented in Table 1.

**Table 1:** Participant demographic and clinical characteristics.

Age (years)	Sex	Heart failure type	Comorbid conditions, n	Comorbid conditions	Prescribed medications, n
71	Female	HFpEF^a^	9	Atrial fibrillation, anemia of chronic disease, chronic kidney disease, diabetes mellitus, hypertension, hyperlipidemia, morbid obesity, and obstructive sleep apnea	16
82	Female	HFrEF^b^	3	Breast cancer, hyperlipidemia, and hypertension	20
67	Female	HFpEF	5	Stroke, pulmonary embolism, atrial fibrillation, and hypertension	7
74	Male	HFpEF	4	Atrial fibrillation, chronic obstructive pulmonary disease, diabetes mellitus, and hypertension	7
69	Female	HFrEF	6	Atrial fibrillation, alcohol abuse, anemia of chronic disease, hypertension, hypothyroidism, and mild liver disease	6
70	Female	HFpEF	8	Coronary artery disease, chronic obstructive pulmonary disease, stroke, diabetes mellitus, hypertension, hypothyroidism, peripheral arterial disease, and obstructive sleep apnea	15
66	Female	HFpEF	9	Chronic obstructive pulmonary disease, coronary artery disease, diabetes mellitus, gastroesophageal reflux disease, hypertension, hyperlipidemia, hypothyroidism, morbid obesity, and obstructive sleep apnea	14
75	Male	HFrEF	6	Coronary artery disease, atrial fibrillation, end-stage renal disease, hypertension, hyperlipidemia, and hypothyroidism	13
85	Male	Unknown	7	Atrial fibrillation, diabetes mellitus, hyperlipidemia, hypertension, hypothyroidism, major depression, and peptic ulcer disease	11
88	Female	HFpEF	7	Coronary artery disease, chronic obstructive pulmonary disease, cerebral venous sinus thrombosis, hypertension, atrial fibrillation, chronic kidney disease, and obstructive sleep apnea	14
85	Female	HFpEF	4	Mitral regurgitation, hypertension, chronic kidney disease, and monoclonal gammopathy of undermined significance	8
76	Male	HFrEF	5	Atrial fibrillation, hypertension, chronic kidney disease, and stroke, and hyperlipidemia	11

^a^HFpEF: heart failure with preserved ejection fraction.

^b^HFrEF: heart failure with reduced ejection fraction.

### CoTI Produced a Codebook Similar to the Senior Investigators’ Codebook

To evaluate whether CoTI could perform thematic analysis similarly to senior investigators, both senior investigators and CoTI extracted clues, identified codes, and developed a codebook with themes, respectively. We then compared the similarity between them.

We first used Instructor to refine instruction prompts that will guide Thematizer (see initial code discovery output in Table S1 in Multimedia Appendix 1). As shown in Table 2, the instruction prompt to extract clues has evolved from an initial, general request for key phrases into a more structured instruction emphasizing direct quotes, contextual completeness, exclusive code assignment, and causal clarity. Similarly, the instruction prompt to generate reasoning advanced to require code-specific, stepwise causal chains, and precise language grounded solely in provided clues.

Then, we applied Thematizer with these refined instruction prompts to extract clues and identify initial codes from all (n=12) transcripts. We calculated Jaccard similarity, precision, recall, and *F*_1_-score between clues extracted by CoTI and those extracted by senior investigators to evaluate clue similarity. We also computed cosine similarity between initial codes generated by CoTI and those identified by senior investigators to measure code similarity. As shown below and in Table 3, our model produced clues and codes more similar to those of senior investigators than did the basic QwQ-32B LLM across most evaluation metrics. Specifically, relatively greater gains were observed in Jaccard score (+7.8%) and precision (+10.9%), with modest gains in *F*_1_-score (+5.4%) and cosine similarity (+4.9%; 0.431 vs 0.411). The response time to extract clues and identify codes was approximately 1 minute per transcript for CoTI and 3 minutes per transcript for the basic QwQ-32B model.

**Table 2:** Optimized instruction prompts by Instructor.

	To extract clues	To generate reasoning
Before optimization	List clues (ie, key phrases, contextual information, semantic and emotional tones, temporal information, symptom descriptions) in the following patient-doctor dialogue that support each given identified topic.	Based on the given clues, generate the reasoning process that supports the identified topics.
After optimization	Extract **direct quotes** from the dialogue that explicitly support each topic, ensuring: 1. **Contextual Completeness & Causal Links**: include specific details that clarify mechanisms or outcomes (eg, *”After doubling the dose, my BP remained at 160/100 despite the doctor’s adjustment”* instead of *”dose increase failed”*). Specify quantitative data, professional feedback, or patient-reported outcomes to strengthen causal relationships. 2. **Exclusive Topic Assignment**: assign each quote to only one topic unless it explicitly addresses multiple themes *simultaneously* (eg, *”My fixed income can’t cover my 12 pills daily”* links both financial burden and polypharmacy). Avoid cross-topic bleeding (eg, *”too many pills”* for cost vs. adherence). 3. **Clarity in Reuse**: for quotes used across topics (eg, religious coping statements), append contextual phrases to clarify relevance (eg, *”I put everything in the Lord’s hands [to cope with stress]”* for psychological themes vs. *”...to accept my medication burden”* for adherence topics). 4. **Causal Precision**: prioritize quotes establishing explicit cause-effect chains (eg, *”The rash from the new pill made me stop taking it”* instead of *”I stopped the pill”*). Specify whether effects are patient-reported, caregiver-observed, or clinically measured. 5. **Discrepancy Framing**: when perspectives conflict, frame quotes within the topic’s context (eg, *”Patient says ‘I take all meds,’ but caregiver notes ‘she skips 3 pills weekly’”* under adherence challenges). Ensure quotes are concise but include sufficient detail to avoid ambiguity and support rigorous reasoning.	For each topic, construct a logical chain connecting clues to the topic by: 1. **Numbered Stepwise Causality**: break down causal pathways into explicit, sequential steps (eg, *”Step 1: Eliquis caused bleeding → Step 2: Fear of overmedication → Step 3: Reduced adherence → Step 4: Uncontrolled condition”*). 2. **Mechanism & Behavioral Impact**: specify *how* each clue leads to outcomes, including patient behavior changes (eg, *”Step 1: High pill count → Step 2: Cognitive overload → Step 3: Missed doses → Step 4: Worsened polypharmacy burden”*; *”Step 1: Financial strain → Step 2: Delayed ER visits → Step 3: Complication escalation”*). 3. **Avoid Assumptions**: explicitly map clues to outcomes using only provided data (eg, *”Step 1: Dose escalation caused nausea → Step 2: Nausea reduced medication intake → Step 3: Suboptimal BP control”* instead of implying indirect links). 4. **Address Contradictions**: explain discrepancies as causal factors (eg, *”Step 1: Patient denies non-adherence → Step 2: Caregiver notes missed doses → Step 3: Conflicting narratives → Step 4: Potential for unmanaged symptoms”*). 5. **Distinct Factor Differentiation**: separate overlapping effects (eg, *”Step 1: High dosage → Step 2: Nausea → Step 3: Reduced adherence”* vs. *”Step 1: Drug interactions → Step 2: Dizziness → Step 3: Fall risk”*). 6. **Actionable Language**: use precise terms like *”triggers,”* *”results in,”* or *”directly causes”* to replace vague phrasing. Ensure reasoning is topic-specific, free of redundancy, and grounded solely in provided clues.

**Table 3:** Evaluation of clue similarity on the heart failure interview transcripts.

	Clue extraction			Relative change (%)
	Basic QwQ-32B LLM	CoTI^a^		
Jaccard similarity	0.374	0.403	+7.75	
Precision	0.496	0.550	+10.87	
Recall	0.635	0.630	–0.79	
*F*_1_-score	0.540	0.569	+5.37	

^a^CoTI: Collaborative Theme Identification Agents.

After generating code for each transcript, we used CodebookGenerator to summarize codes across all transcripts into a structured codebook with themes (see outputs in Table S2 in Multimedia Appendix 1). We calculated the cosine similarity between the CoTI-generated and senior-generated themes to evaluate theme similarity. CoTI-generated themes were more similar to the senior investigator’s than those produced by traditional NLP topic modeling methods (LDA: 0.391; Top2Vec: 0.377; BERTopic: 0.266) or the basic QwQ-32B LLM (+22.24%; 0.621 vs 0.508). The codebook generation time was approximately 10 seconds for CoTI and 2 minutes for the basic QwQ-32B model. Textbox 1 shows the overlap between the CoTI- and senior-generated themes. CoTI successfully captured several major themes identified by senior investigators, including adverse drug effects, psychological distress, burden from the number of medications, and burden from the cost of medication.

**Comparison of codebook with themes developed by senior investigators and Collaborative Theme Identification Agents (CoTI; without junior investigators) on the heart failure interview transcripts.**

**Senior investigator**

- Problems in logistics
- Impact from the patient-doctor relations

**Overlap**

- Adverse drug effects
- Psychological distress
- Burden from the number of medications
- Burden from the cost of medications

**CoTI**

- Medication adherence and management challenges
- Desire for simplified medication regimen
- Perceived effectiveness of medications
- Patient-doctor relationship and communication

### CoTI Alone Was More Similar to Senior Investigators Than Junior Investigators or CoTI-Junior Collaboration

After verifying that CoTI can generate themes more similar to those of senior investigators than other NLP models or the basic LLM, we conducted an exploratory human-AI collaboration experiment to examine whether a human (particularly one with low expertise, such as a junior investigator) could further refine CoTI-generated outputs. The purpose of this experiment was not to compare CoTI with junior investigators performing the full thematic analysis workflow from scratch. Instead, because CoTI is designed to improve the efficiency of thematic analysis by generating clues, initial codes, and themes for human review, we evaluated whether junior investigators could use CoTI-generated outputs as a starting point and refine them.

We compared three settings (Table 4): (1) junior investigator alone, where junior investigators independently identified codes for a subset of assigned transcripts; (2) CoTI alone, where our model extracted clues, identified codes, and generated themes without junior investigators’ feedback for all transcripts; and (3) CoTI+junior investigator, where junior investigators used the CoTI application to review model-generated clues and codes, and provided feedback to CoTI to refine its outputs for each assigned transcript. In all settings, clues, codes, and themes provided by senior investigators served as the reference standard. Since we considered codes as the final analytic output of each transcript, junior investigators were only responsible for manually identifying codes and did not perform clue extraction. Additionally, because the themes can be generated only after considering codes across all transcripts and junior investigators were assigned only a subset of transcripts, they could not construct a complete codebook with themes. Therefore, the evaluation focused on clue extraction for the CoTI alone and CoTI+junior investigator settings, and on code identification for all 3 settings.

**Table 4:** Four different settings to perform thematic analysis on the heart failure interview transcripts.

Setting	Interview transcripts to process (N=12), n	Extract clues	Identify codes	Generate themes
Junior investigator alone	7	No	Yes	No
CoTI^a^ alone	12	Yes	Yes	Yes
CoTI+junior investigator	7	Yes	Yes	No
Senior investigators only (reference standard)	12	Yes	Yes	Yes

^a^CoTI: Collaborative Theme Identification Agents.

To evaluate whether junior investigators’ feedback could help CoTI to extract clues that are more similar to those of the senior investigators, we compared clue similarity between CoTI alone and the senior investigators, and between CoTI with junior investigators’ feedback and the senior investigators. Among the evaluation metrics, we prioritized recall as the most meaningful evaluation metric since higher recall indicates that the model successfully retrieves a large proportion of relevant clues that were identified by senior investigators, which is critical for ensuring that subsequent code identification is grounded in a sufficiently rich evidence base. Although precision reflects the accuracy of extracted clues, occasional inclusion of clues not identified by senior investigators may be less damaging because qualitative thematic analysis is inherently subjective; additional identified results may also be valid even if they are not present in the reference standard. As shown in Figure 4A, CoTI with junior investigators’ feedback resulted in small or modest recall improvements in many transcripts. While precision was not the primary metric, it offers complementary insight into the correctness of extracted clues. As shown in Figure 4B, CoTI with junior investigators’ feedback generally yielded lower precision compared to CoTI alone. Even in the few cases where precision improved after junior investigators’ feedback, such as transcripts 3 and 7, the gains were marginal. These results suggest that CoTI alone already provides strong clue similarity, and junior investigators’ feedback brings limited or even negative impact on improving clue similarity with senior investigators. One possible hypothesis is that the gap between CoTI and senior investigators may involve domain-specific understanding that junior investigators also lack. As a result, junior investigators’ feedback may not sufficiently refine the model’s outputs. Another possible hypothesis is that junior investigators may have exhibited automation bias, a tendency to rely on outputs from CoTI, and this overreliance could have reduced their critical engagement with the model’s outputs.

We continued evaluating the impact of junior investigators’ feedback for the task of code identification. As shown in Figure 4C, codes identified by both CoTI alone and CoTI with junior investigators’ feedback achieved higher similarity to senior investigators than codes manually identified by junior investigators in many cases. Notably, for some cases, incorporating junior investigators’ feedback to CoTI led to identified codes that were more similar to those of senior investigators, as seen in transcripts 2, 3, 4, and 8, where CoTI alone achieved moderate theme similarity scores (approximately between 0.35 and 0.40). However, when CoTI alone already achieved strong code similarity to senior investigators (cosine similarity ≥ 0.45), incorporating junior investigators’ feedback tended to reduce that similarity, as observed in transcripts 5, 6, and 10. Compared to the clue extraction task, junior investigators’ feedback appeared more helpful in supporting the code identification task, suggesting junior investigators may be more adept at higher-level interpretation than at intermediate clue extraction.

The marginal benefit of junior investigators’ feedback to CoTI might be due to junior investigators’ perception of AI. To explore this possibility, we collected their perceptions of CoTI. As illustrated in Figure 5A, subjective ratings were generally high across all junior investigators, indicating strong approval of CoTI’s accuracy, relevance, trustworthiness, and overall satisfaction. These favorable perceptions suggest that junior investigators may have felt less need to critically revise or question CoTI’s outputs, thereby reducing the additive value of AI-human collaboration. However, we noticed several exceptions. Investigator D rated CoTI as missing codes in every assigned transcript, resulting in a code comprehensiveness score of 0%. This is particularly striking given that D simultaneously gave perfect trust (100%) and top scores across most other dimensions. To better understand this contradiction, we analyzed D’s performance in the manual code identification task. D achieved a code similarity of 0.41, higher than investigators A and C, though slightly lower than CoTI alone (code similarity=0.45). Additionally, D identified a total of 47 codes across assigned interviews, compared to 41 themes identified by CoTI. These results suggested that investigator D was particularly context-sensitive and may have maintained a high bar for code completeness. Although D trusted CoTI’s identified codes, D likely expected CoTI not only to identify obvious codes but also to capture more implicit ones. In addition, collaboration between D and CoTI led to small gains in clue similarity compared to CoTI alone, which indicated that even for a higher standard evaluator, collaboration with AI offered marginal but observable benefits. Another notable exception was investigator A, whose performance in code identification was comparatively weaker than investigators B and D. Moreover, CoTI alone achieved higher code similarity than A’s individual performance, and collaboration between A and CoTI improved clue similarity compared to CoTI alone. Nevertheless, investigator A reported lower trust in CoTI compared with other investigators, indicating that A’s confidence in AI remained low even when CoTI outperformed A’s own performance and the collaboration yielded measurable benefits.

**Figure 4:**
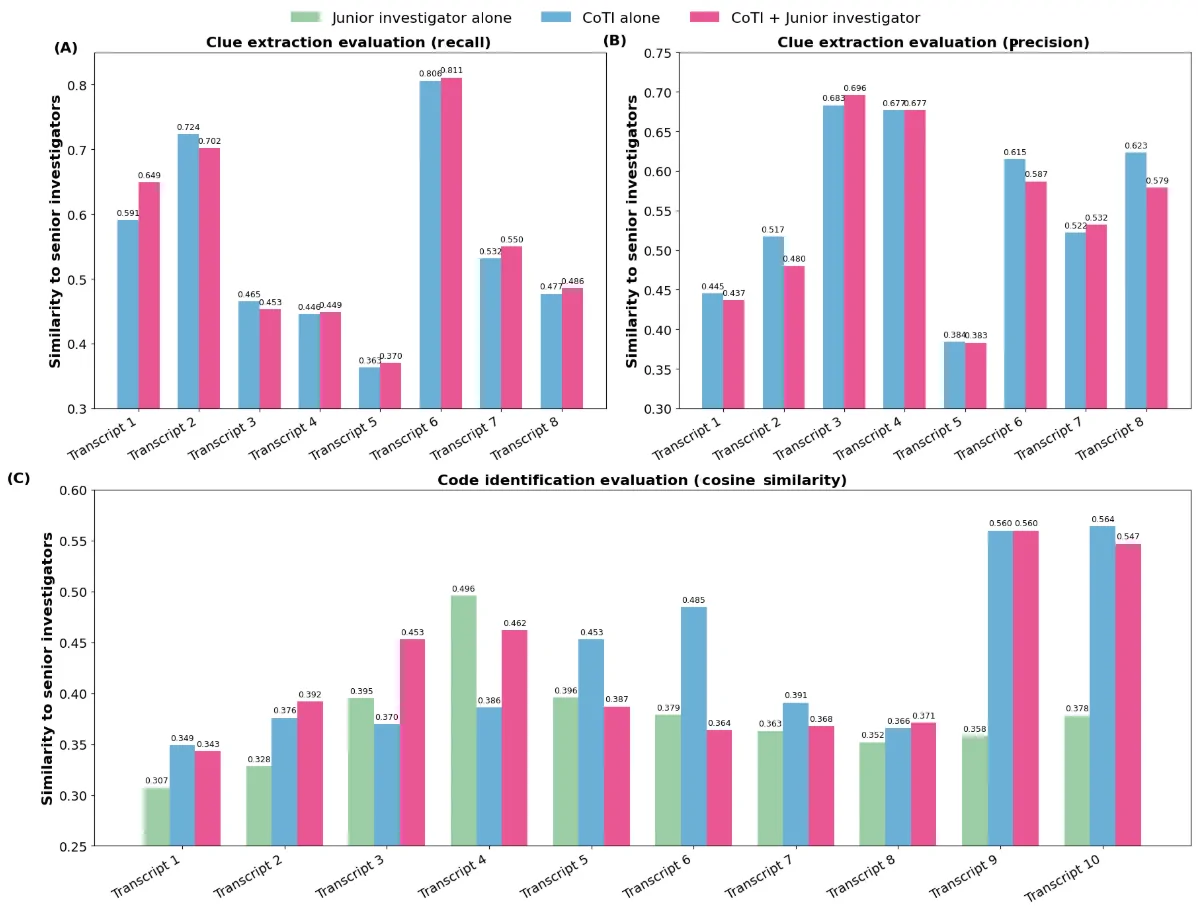
Evaluation of clue extraction and code identification across interviews. (A) Recall of extracted clues for Collaborative Theme Identification Agents (CoTI)–only and CoTI+human settings, evaluated against clues identified by senior investigators. (B) Precision of extracted clues for CoTI-only and CoTI+human settings, evaluated against clues identified by senior investigators. (C) Cosine similarity between codes generated by human-only, CoTI-only, or CoTI+human, compared to senior investigators’ identified codes (reference standard).

**Figure 5:**
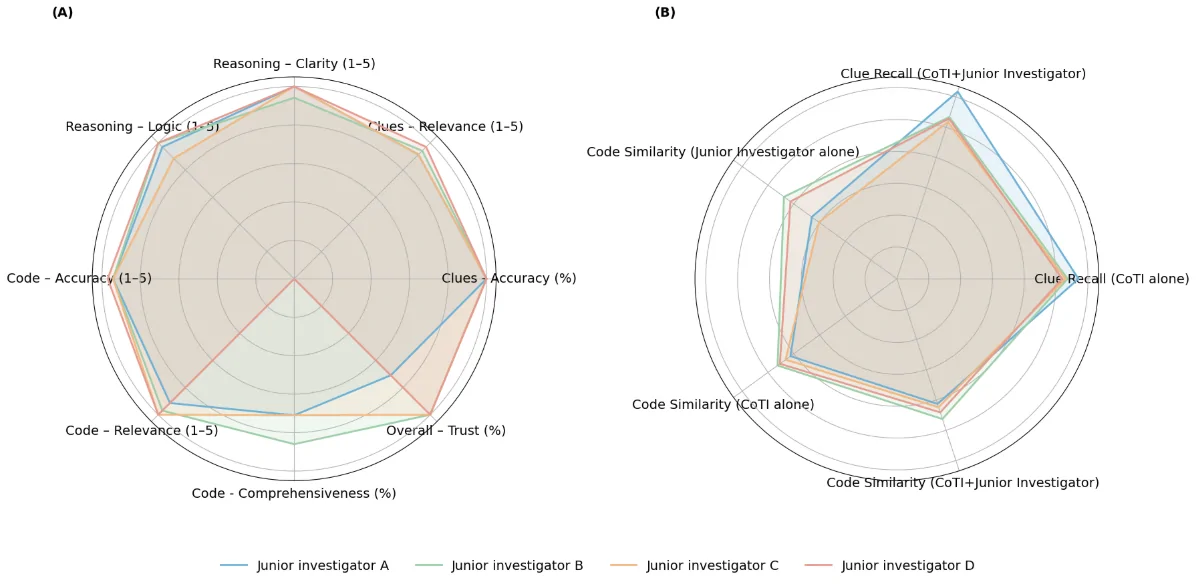
Subjective survey and objective evaluations by junior investigators. (A) This spider chart displays subjective perceptions from 4 junior investigators, A-D, regarding Collaborative Theme Identification Agents (CoTI). Subjective measures include ratings of clue accuracy and relevance, reasoning clarity and logic, code accuracy, relevance, and comprehensiveness, as well as trust and overall satisfaction. (B) This spider chart displays objective performance by junior investigators and CoTI, including clue recall (CoTI alone), clue recall (CoTI+junior investigator), code cosine similarity (junior investigator alone), code cosine similarity (CoTI alone), and code cosine similarity (CoTI+junior investigator).

### Ablation Study Highlights Components’ Contributions to CoTI Performance

Aiming to understand the contributions of each component within our model, we conducted an ablation study (Table 5). The study began with a GPT-4o-mini model, which served as the baseline. In this setting, GPT-4o-mini performed thematic analysis only one time for each transcript (total 12). Introducing Instructor, an agent designed to iteratively refine instruction prompts, improved the similarity of extracted clues (Jaccard similarity: +14.3%; precision: +24.3%; recall: +2.7%; *F*_1_-score: +11.0%). These results highlighted the critical role of high-quality, tailored instruction prompts in improving CoTI’s ability to extract clues that were more similar to those extracted by senior investigators. Subsequently, we applied a multirun aggregation strategy, which repeated the code identification, including clue extraction, 3 times and retained the union of the resulting outputs. This strategy further improved performance, yielding relatively greater gains in clue similarity (Jaccard similarity: +17.5%; recall: +18.7%; *F*_1_-score: +12.5%) with modest improvements in code similarity (cosine similarity: +0.9%). These findings indicated that multiple code identification passes allowed our model to capture a broader range of codes and supporting clues, including those that may be missed in a single run due to the randomness in LLM outputs.

**Table 5:** Overview of Collaborative Theme Identification Agents (CoTI) components and additive contributions to the performance.

	Clue extraction					Code identification		Response time for each transcript
	Jaccard similarity (relative change, %)^a^	Precision (relative change, %)^a^	Recall (relative change, %)^a^	*F*_1_-score (relative change, %)^a^	Cosine similarity (relative change, %)^a^		Time (seconds)	
GPT-4o-mini	0.300 (N/A^b^)	0.441 (N/A)	0.517 (N/A)	0.456 (N/A)	0.433 (N/A)		~8	
GPT-4o-mini with Instructor	0.343 (+14.33)	0.548 (+24.26)	0.531 (+2.71)	0.506 (+10.96)	0.427 (–1.39)		~20	
CoTI (GPT-4o-mini with Instructor and multirun aggregation)	0.403 (+17.49)	0.550 (+0.36)	0.630 (+18.64)	0.569 (+12.45)	0.431 (+0.94)		~60	

^a^Values indicate the relative change (%) compared with the preceding model configuration.

^b^N/A: not applicable.

### Generalizability of CoTI on External Datasets

To further evaluate the generalizability of CoTI, we applied our framework to several different health care qualitative datasets, including the health system’s response to COVID-19 in Sierra Leone, which comprised 21 interview transcripts [25], and the family carers’ strategies when a family member with dementia was agitated, which comprised 18 interview transcripts [29]. Table S4 in Multimedia Appendix 1 shows the computational evaluations among these external datasets. Tables S5 and S6 in Multimedia Appendix 1 show overlaps between the themes generated by our model and by experts. Across these external datasets, CoTI achieved higher clue similarity than theme similarity, indicating that it identified relevant supporting excerpts more effectively than it reproduced expert-level themes. This suggests that human researcher review remains necessary to interpret and validate generated themes.

## Discussion

Our study demonstrates the efficiency of leveraging multiagent LLMs to support thematic analysis in qualitative research. By integrating prompt engineering with multiagent collaboration, our proposed framework successfully extracted supporting excerpts, identified initial codes, and developed a codebook with themes through an automated process. Beyond its application in heart failure qualitative research, our framework has demonstrated its efficiency in other domains, such as COVID-19–related and Alzheimer-related studies. In addition, our developed CoTI interactive application allows human researchers to rapidly review model-generated outputs, locate key transcript sections by searching keywords derived from model-extracted supporting excerpts, and provide feedback to refine the outputs. This interactive design facilitates human-AI collaboration and may help researchers more efficiently gain insights into patients’ experiences, thereby facilitating qualitative research. However, our preliminary experiment found that collaboration between CoTI and junior investigators yielded marginal improvements over CoTI alone in the heart failure study.

The computational evaluations showed that the themes generated by CoTI alone were more similar to those identified by senior investigators than those generated by junior investigators working independently or by baseline models. To further examine the quality of these themes, we incorporated an expert qualitative appraisal as a complementary assessment. The expert identified overlap among the themes “medication burden,” “psychological and emotional impact of medications,” and “side effects and their perception.” In particular, the expert considered “side effects and their perception” unnecessary as a separate theme because these experiences can be integrated within either medication burden or the psychological and emotional impact of medications. Similarly, the expert noted that “desire for simplified medication regimen” can be integrated into “medication adherence and management challenges.” These observations suggested that CoTI may separate related experiences into multiple themes, resulting in thematic distinctiveness that is less clearly differentiated. This qualitative appraisal aligns with our computational evaluation, which indicated relatively modest theme distinctiveness (0.473).

Next, we compared CoTI-generated themes with the reference themes developed by senior investigators to identify where CoTI missed or partially distorted the reference interpretations. One missed example was the reference theme “problems in logistics.” In senior investigators' generated interpretations, this theme referred not only to medication-taking behavior but also to broader practical barriers, including transportation difficulties, obtaining medications, coordinating refills, identifying the correct pharmacy, and relying on family members or caregivers to complete multiple steps in the medication process. CoTI did not generate an equivalent theme. Instead, it generated the theme “medication adherence and management challenges,” which focused more narrowly on patients’ difficulties with medication adherence, including forgetfulness, confusion, overdosing, and the need for structured support systems. One distorted example involved the reference theme “impact from patient-doctor relations.” The senior investigators' generated theme captured a complex pattern that included both trust and dependency on physicians, as well as skepticism toward pharmaceutical companies and insurance systems. CoTI generated a related theme, “patient-doctor relationship and communication,” but this theme emphasized communication gaps and patient education rather than the broader trust-mistrust tension captured in the reference interpretations. Another partially distorted example was CoTI’s theme “desire for simplified medication regimen.” Although the supporting excerpts for this theme overlapped with those extracted by senior investigators, the senior investigators interpreted these excerpts as part of the broader theme “burden from the number of medications.” In contrast, CoTI considered patients’ preferences for a simplified or reduced medication regimen as a separate theme, rather than integrating them into medication burden. CoTI also generated a theme “perceived effectiveness of medications,” which was plausible but not supported by the reference interpretations. Although some patients discussed whether their medications were working, the senior investigators’ generated themes did not identify it as a separate theme. This suggested that CoTI may sometimes identify some less important patterns as distinct themes even when human researchers interpret them as secondary or insufficiently central to the overall research question. Together, these examples suggest that while CoTI can identify relevant semantic content, it may sometimes reorganize these patterns into themes that are more general, more familiar, or less central to the research question than those developed by human researchers. These findings indicate that CoTI can serve as an efficient tool to support thematic analysis, but its outputs still require careful human review and interpretation. This conclusion is consistent with Shanwetter et al [15], who emphasized that LLMs should complement rather than replace human analysis, particularly when identifying structural themes.

Our study also provides preliminary insights into the potential role of CoTI in supporting human-AI collaboration in thematic analysis. First, CoTI alone produced codes more similar to senior investigators than junior investigators. This highlights CoTI’s potential as a reliable tool for thematic analysis, particularly when senior investigators are unavailable. Second, collaboration with junior investigators did not consistently improve clue or code similarity to senior investigators. While human-AI collaboration is often assumed to enhance AI outputs, our results show that the effectiveness is marginal. Specifically, when CoTI alone code similarity was moderate to senior investigators, junior investigators’ feedback could help refine codes and enhance similarity. However, when CoTI alone already performed strongly, such feedback often degraded code similarity. In addition, CoTI alone extracted clues that often achieved higher recall and precision; however, incorporating junior investigators’ feedback sometimes made clue similarity decrease. Survey analyses indicated that the marginal benefits of collaboration may stem from junior investigators’ overreliance on CoTI’s outputs, which could cause automation bias and reduce their independent critical thinking.

Our study has several limitations. First, the human-AI collaboration was exploratory. We recruited only 4 junior investigators, who analyzed selected subsets of transcripts and did not complete the full thematic analysis workflow, including excerpt extraction, code identification, and theme generation through a consensus or adjudication process. Therefore, our findings only provided preliminary evidence that CoTI-generated outputs were more similar to the senior investigators’ generated reference standard than those generated by the participating junior investigators. Future studies should evaluate both CoTI alone and CoTI-assisted human-AI collaboration against rigorous human-led thematic analysis workflows involving multiple trained coders, consensus coding, and adjudication, thereby providing a more rigorous assessment of CoTI’s effectiveness and the potential benefits of human-AI collaboration in thematic analysis. Second, the human-AI collaboration was conducted on a single case study of heart failure interviews, which limits the generalizability of our findings on human-AI collaboration in thematic analysis. Further validation across other research contexts is needed to establish broader generalizability. Moreover, the potential impact of incorporating feedback from senior investigators into CoTI was not evaluated, which may limit understanding of how senior investigators’ input could further enhance our model performance. Another limitation is that our interpretation of automation bias was based on a small exploratory survey of junior investigators’ perceptions of CoTI. Although the survey results suggested that the high trust in CoTI may reduce junior investigators’ tendency to critically revise AI-generated outputs, these findings remain preliminary because of the small sample size and reliance on self-reported perceptions. Moreover, alternative explanations cannot be excluded, such as limited qualitative analysis experience among junior investigators and lower quality of junior investigators’ feedback. Therefore, we cannot consider automation bias or other factors as the definitive explanations for the limited additive benefit of junior investigators’ involvement.

In all, CoTI provides preliminary evidence that multiagent LLMs can replicate key phases of qualitative thematic analysis while maintaining human involvement through review and refinement. In addition, our study suggests that the value of LLMs in qualitative research lies not in replacing researchers, but in expanding their capacity to analyze larger and more complex datasets efficiently while maintaining interpretive oversight. Future research should focus not only on improving model performance, but also on designing collaborative workflows that preserve reflexivity and critical interpretation.

## Appendix

Multimedia Appendix 1 Supplementary tables and detailed prompt design of the large language model workflow.

## Abbreviations

CoTI: Collaborative Theme Identification Agents
IRB: institutional review board
LDA: latent Dirichlet allocation
LLM: large language model
NLP: natural language processing
QUEST: quality, understanding, expression, safety, and trust

## Data Availability

The interview transcripts about patients with heart failure cannot be made publicly available due to privacy concerns. For interview transcripts about COVID-19, please see Open Science Framework [25,30]. For interview transcripts about Alzheimer-related topics, please see Mendeley Data [28,30]. The code used in this study is available on GitHub [32].
