# Supplementary material for: Automated Health Care Thematic Analysis Using a Multiagent Large Language Model: Algorithm Development and Evaluation Study
Source: J Med Internet Res. 2026 Sep 30;28:e90872. doi: 10.2196/90872 (PMC13669709; doi:10.2196/90872)
Supplement: Supplementary file 1 [file jmir_v28i1e90872_app1.docx]

# **Supplementary**

**Section A. Prompts**

**Textbox S1. Prompt for initial code discovery**

**System:**

\### Background ###

Heart failure patients' perception of medication intensity is a complex experience influenced by factors beyond dosage, including side effects, treatment burden, psychological impact, quality of life, and cost. This perception is shaped by patient characteristics such as age, disease severity, comorbidities, gender, and socioeconomic status. Understanding these perceptions, along with patients' beliefs about their medications, is crucial for improving adherence and tailoring treatment strategies to enhance their quality of life. Ultimately, a patient-centered approach that involves effective communication and shared decision-making is essential in managing heart failure medication regimens.

You are a qualitative research expert tasked with identifying topics of patient-doctor dialogues. These patients are diagnosed with heart failure. You are trying to understand patient's perception of the intensity of heart failure medications.

Your task:

- Identify important topics for the given dialogue (there may be more than one).

- For each identified topic, provide clues and reasoning to explain the connection.

Clues must:

- Be direct quotes from the dialogue (no summarization or interpretation).

- Be brief but contextually complete.

- Highlight key phrases, contextual information, emotional tones, or symptoms related to the topic.

Reasoning must:

- Links the clues directly to the topic.

- Explains the logical connection between the clues and topic.

- Avoids adding external context or information not present in the clues.

**User:**

Your task is to identify important topics for the given interview.

Each topic should be concise, meaningful, and specific. Avoid combining distinct ideas or using vague terms.

Step 1 Extract CLUES.

Step 2 Generate REASONING.

Step 3 Identify TOPICS: Based on the dialogue, clues, and reasoning, identify all applicable topics.

\### Output Format ###

For EACH identified topic, provide the following EXACTLY:

Identify topic: [Insert topic here]

Clues (max 200 words): [Insert clues here]

Reasoning (max 150 words): [Insert reasoning here]

Dialogue: {dialogue}

**Textbox S2. Prompt for initial clue instruction (*Clue-LLM*)**

**System:**

You are a qualitative research expert with extensive experience analyzing patient-doctor dialogues. These patients are diagnosed with heart failure. You are trying to understand patient's perception of the intensity of heart failure medications. Your task is to extract key clues (limit to 200 words) directly from original dialogues supporting each given identified topic.

Clues must:

- Be direct quotes from the dialogue (no summarization/interpretation/explanation).

- Be brief but contextually complete.

- Highlight key phrases, contextual information, emotional tones, or symptoms related to the topic.

**User:**

List clues (i.e. key phrases, contextual information, semantic and emotional tones, temporal information, symptom descriptions) in the following patient-doctor dialogue that support each given identified topic.

Dialogue: {dialogue}

Topics: {topics}

\### Output Format EXACTLY following ###

Topic: clues

**Textbox S3. Prompt for initial reasoning instruction (*Reasoning-LLM*)**

**System:**

You are a qualitative research expert tasked with analyzing patient-doctor dialogues. These patients are diagnosed with heart failure. You are trying to understand patient's perception of the intensity of heart failure medications. Your goal is to provide a clear and concise reasoning process (limit to 150 words) based on provided clues to explain each corresponding identified topic.

Ensure your reasoning:

- Links the clues directly to the topic.

- Explains the logical connection between the clues and topic.

- Avoids adding external context or information not present in the clues.

**User:**

Based on the given clues, generate the reasoning process that supports the identified topics.

Clues: {clues}

Topics: {topics}

\### Output Format EXACTLY following ###

Topic: reasoning

**Textbox S4. Prompt for evaluation (*Evaluation-LLM*)**

**System:**

You are an evaluation expert tasked with analyzing a BATCH of patient-doctor dialogue clue-reasoning-topic pairs. These patients are diagnosed with heart failure, and the focus is on understanding their perception of the intensity of heart failure medications.

Your tasks are as follows:

1. Evaluate each Clues-Reasoning-Topic pair for Clue Quality and Reasoning Quality.

2. Provide feedback on both the relevance and completeness of the clues, and the logical coherence of the reasoning.

3. Identify common issues across all pairs in clue and reasoning generation.

4. Suggest improvements to the clue and reasoning prompts based on recurring patterns of errors.

**User:**

Clues-Reasoning-Topic Pair {i + 1}

**Clues:** {clues}

**Reasoning:** {reasoning}

**Topics:** {topics}

\### Evaluation Task ###

For the above Clues-Reasoning-Topic pair:

1. **Clue Quality:** Evaluate the clues based on the following:

- How relevant and accurate are the clues in supporting the topic(s)?

- Are the clues complete (include all key information) and free of irrelevant details?

2. **Reasoning Quality:** Assess the reasoning based on the following:

- Does the reasoning logically connect the clues to the topic(s)?

- Are there any gaps or missing logic in the reasoning process?

- Is the reasoning concise and free of unnecessary content?

\### Aggregate Feedback Task ###

Based on your evaluation of all the Clues-Reasoning-Topic pairs, provide in the following format EXACTLY following:

\### Aggregate Feedback Task ###

**Common Issues:**

- **Clue Generation:** Identify recurring problems in the generated clues (e.g., missing context, irrelevant clues).

- **Reasoning Generation:** Highlight frequent issues in reasoning (e.g., logical gaps, weak connections between clues and topics).

**Suggestions for Improvement:**

- **Clue Prompt:** Propose specific improvements to the clue generation prompt.

- **Reasoning Prompt:** Recommend actionable enhancements to the reasoning generation prompt.

**Textbox S5. Prompt for optimization (*Optimization-LLM*)**

**System:**

You are part of an optimization system that improves text. You will be asked to creatively and critically improve the clue prompt and reasoning prompt (instructions). You will receive some feedback, and use the feedback to improve both clue and reasoning prompts simultaneously. The feedback may be noisy, identify what is important and what is correct. Pay attention to the role description of the clue and reasoning prompts (instructions), and the context in which it is used.

**User:**

You are tasked with improving two prompts based on provided feedback.

\### Task Description ###

Given the following feedback, improve both the **Clue Prompt** and the **Reasoning Prompt** simultaneously to address the issues and suggestions provided:

1. The **Clue Prompt** should:

- Guide the user/system to extract relevant, precise, and contextually complete clues directly from the dialogue.

- Ensure the clues are accurate quotes, avoid irrelevant or incomplete clues, and incorporate missing elements identified in the feedback.

- Focus on ensuring clarity and usability of the prompt.

2. The **Reasoning Prompt** should:

- Guide the user/system to logically and effectively connect the clues to the identified topics."

- Ensure the reasoning structure is clear, addresses logical gaps, and builds a strong link between the clues and topics."

- Incorporate improvements to reasoning clarity and structure as per the feedback.

\### DO NOT VIOLATE THE FOLLOWING SYSTEM RULES ###

These are foundational instructions that must NEVER be contradicted or weakened.

[CLUE RULES]

Be direct quotes from the dialogue (no summarization/interpretation/explanation).

Quotes must be brief but contextually complete.

[REASONING RULES]

Use only the provided clues.

Do not introduce external information or assumptions.

\### Provided Inputs ###

**Feedback:**{feedback}

**Current Clue Prompt:**{clue_prompt}

**Current Reasoning Prompt:**{reasoning_prompt}

\### Output Instructions ###

You MUST provide your improved prompts formatted as follows:

- For the clue prompt: <IMPROVED_CLUE_PROMPT> your improved clue prompt text </IMPROVED_CLUE_PROMPT>

- For the reasoning prompt: <IMPROVED_REASONING_PROMPT> your improved reasoning prompt text </IMPROVED_REASONING_PROMPT>

The text provided between these tags will directly replace the current prompts, so ensure your improvements are complete, clear, and directly address the feedback provided.

**Textbox S6. Prompt for code identification (*Thematizer*)**

**System:**

\### Background ###

Heart failure patients' perception of medication intensity is a complex experience influenced by factors beyond dosage, including side effects, treatment burden, psychological impact, quality of life, and cost. This perception is shaped by patient characteristics such as age, disease severity, comorbidities, gender, and socioeconomic status. Understanding these perceptions, along with patients' beliefs about their medications, is crucial for improving adherence and tailoring treatment strategies to enhance their quality of life. Ultimately, a patient-centered approach that involves effective communication and shared decision-making is essential in managing heart failure medication regimens.

You are a qualitative research expert tasked with identifying topics of patient-doctor dialogues. These patients are diagnosed with heart failure. You are trying to understand patient's perception of the intensity of heart failure medications.

Your task:

- Identify **all applicable topics** for the given dialogue (there may be more than one).

- For each identified topic, provide clues and reasoning to explain the connection.

Clues must:

- Be direct quotes from the dialogue (no summarization or interpretation).

- Be brief but contextually complete.

- Highlight key phrases, contextual information, emotional tones, or symptoms related to the topic.

Reasoning must:

- Links the clues directly to the topic.

- Explains the logical connection between the clues and topic.

- Avoids adding external context or information not present in the clues.

**User:**

Your task is to identify ALL applicable topics for the given dialogue. Topics should be about patient's perception of the intensity of heart failure medications. There may be multiple topics, so ensure you capture each distinct one.

Step 1 Extract CLUES: {optimized_clue_prompt}

Step 2 Generate REASONING: {optimized_reasoning_prompt}

Step 3 Identify TOPICS: Based on the dialogue, clues, and reasoning, identify all applicable topics.

\### Output Format ###

For EACH identified topic, provide the following EXACTLY:

Identify topic: [Insert topic here]

Clues (max 200 words): [Insert clues here]

Reasoning (max 150 words): [Insert reasoning here]

Dialogue: {dialogue}

**Textbox S7. Prompt for aggregation**

**System:**

You are an AI assistant analyzing topic identification outputs to aggregate results effectively.

**User:**

You are analyzing topic identification outputs from multiple analyses of the same patient-doctor dialogue.

\### Your Goal ###

Aggregate all topics found across multiple outputs. If a topic appears in multiple outputs:

- Merge all its associated clues (without modification).

- Concisely summarize the reasoning.

- Choose the best topic name from the outputs.

For unique topics (appearing only once):

- Keep them as they are.

\### Output Format ###

Provide your results in the following format for EACH topic:

Topic: [Insert best topic name]

Clues (max 200 words): [Insert aggregated clues]

Reasoning (max 150 words): [Insert summarized reasoning]

Output 1: {result_1}

Output 2: {result_2}

Output 3: {result_3}

**Textbox S8. Prompt for theme generation (*CodebookGenerator*)**

**System:**

You are a qualitative research expert assisting in developing a thematic codebook from structured interview results.

Each entry consists of:

- **Topic**: an initial code identified in one interview.

- **Clues**: direct quotes from the interview.

- **Reasoning**: why this topic is relevant or meaningful.

\### Your task ###

- Review all topic-clue-reasoning triples.

- Merge them into distinct, high-quality themes.

Each theme in the codebook should include:

- `theme_name`: The name of the higher-level concept

- `description`: A short explanation of what this theme captures and why the grouped topics fit

- `original_topics`: The list of topics it covers

- `representative_clues`: A few relevant supporting quotes

Only merge topics when there is a strong conceptual overlap. Be precise and avoid redundancy.

\### Instructions for Codebook Formation ###

1. **Group original topics into broader, higher-level themes** based on a **single, clearly identifiable shared key concept**.

2. **Each original topic must belong to exactly one higher-level theme**. Avoid overlap or duplication.

3. **Do not merge topics solely based on vague thematic similarity**. Merging must be grounded in a specific, shared concept.

4. **Higher-level themes must be mutually exclusive**, covering distinct conceptual territories.

5. If an original topic does not share a strong conceptual basis with any others, treat it as its own higher-level theme.

**User:**

Below is a JSON file containing multiple identified topics with extracted clues and generated reasoning.

**Your task is to create a robust and conceptually sound codebook.** This is a crucial step in thematic analysis for organizing and synthesizing qualitative data.

\### Input JSON: {original json file}

**Section B. Survey to understand junior investigators’ perception about CoTI’s outputs**

| **Output** | **Dimension** | **Question** | **Scale** |
| --- | --- | --- | --- |
| Clues | Accuracy | Is this quote directly from the transcript and correctly cited? | Yes/No |
| Clues | Relevance | Is this quote clearly related to the identified code? | 1-5 |
| Reasoning | Clarity | Is the explanation easy to follow? | 1-5 |
| Reasoning | Reasoning | Does it logically connect the clues to the code? | 1-5 |
| Code | Accuracy | Is the identified code correct based on the context? | 1-5 |
| Code | Relevance | Is the identified code related about patient perceptions of the intensity of HF medications? | 1-5 |
| Code | Comprehensiveness | Did the model miss other major codes? | Yes/No (with comment) |
| Overall | Trust | Would you trust this app to assist in annotation? | Yes/No  (with comment) |
| Overall | Satisfaction | Are you satisfied with this app? | 1-5 |

**Section C. Themes Analysis Results**

**Table S1. Initial code discovery**

| **Interview** | **Initial Codes** | **Clues** | **Reasoning** |
| --- | --- | --- | --- |
| 11 | Perceived Efficacy of Entresto | "I guess it is."  "No, I don’t [feel much different]." "If the doctor stopped the medication, is it okay?  "Well, I guess, they would know best, I guess. I don’t know." | The interviewee repeatedly expresses uncertainty about Entresto’s effectiveness, stating no noticeable symptom improvement and deferring to the doctor’s judgment. This highlights her perceived lack of efficacy, a key aspect of medication intensity perception. |
| 11 | Dosage Intensity and Side Effects | "From forty-nine to ninety-seven [mg]. That was a big jump."  "The blood pressure increased more." "Entresto makes her drowsy."  "They had her on a low dose at first… then it went from what, forty something to ninety something. There was a big jump." | The dosage increase caused adverse outcomes (e.g., rising blood pressure) and side effects like drowsiness, directly linking dosage intensity to negative physical experiences and patient concern. |
| 11 | Medication Burden from Polypharmacy | "She takes too much medication. It gets confusing."  "Seven at one time in the morning, three or four in the afternoon, and five at night."  "You’ve got to take the vitamins… aspirin… iron tablets."  "I take too many." | The daughter and patient explicitly state that the high number of medications (polypharmacy) causes confusion, cognitive burden, and overwhelm, a core theme of perceived medication intensity. |
| 11 | Cost Concerns of Entresto | "The deductible… $7,500.00… it’s crazy."  "The only thing, back when- because he had me on a low dose… the cost?" "Entresto… the only thing [financial]… a big jump." | The patient and daughter emphasize the financial strain of Entresto’s high deductible and dosage changes, reflecting cost as a significant component of perceived medication intensity. |
| 11 | Impact of Side Effects on Daily Function | "The Entresto makes her drowsy." "Three things [pain meds] that was taking her to sleepy, drowsy mode." "I don’t have any energy to do anything… half of the day is gone." "It blurs my vision sometimes." | Side effects like excessive drowsiness and vision issues directly disrupt the patient’s daily functioning, illustrating how perceived medication intensity affects quality of life. |
| 11 | Adherence Challenges Due to Complexity and Fatigue | "Sometimes I forget."  "It depends on my situation… sometimes I do, but then if it says eat first, then sometimes I don’t want to be eating."  "She may skip the afternoon… take the night time before she goes to bed."  "The only thing hard for her is to prepare the meals… she has to have the walker." | The patient’s forgetfulness, reliance on caregivers for organization, and physical/mental fatigue (from cancer treatment and mobility issues) create barriers to adherence, underscoring medication regimen complexity as a key perception factor. |
| 12 | High medication burden due to quantity and frequency | "I think I’m over taking them, too much medicine, but that’s just me."  "I don’t think I take that or not."  "About eight or nine in the morning… probably about six at night."  "The only problem I have is being on dialysis and stuff, my bedroom is stack full of the boxes."  "I just … I put everything in the Lord’s hands. The Lord’s taking care of everything." | The patient explicitly states feeling overwhelmed by the number of medications, noting the high quantity (8–9 pills in the morning, 6 at night) and the physical clutter of medical supplies. His acknowledgment of feeling “overmedicated” and reliance on religious coping to manage the burden directly links to his perception of medication intensity as physically and emotionally taxing. |
| 12 | Financial burden of medication costs and hospitalizations | "Brilinta’s, I think that’s $300-and some for three-month supply."  "I probably spend close to $300 a month on medication."  "every time I go to the emergency room, I think it’s … 250 out of me right there. And then if I take the ambulance, that’s another 250."  "it ain't long and then you got $3,000, $4,000, $5,000 worth of debt."  "you work all your life and then to have something like this or something like that … it’s depressing." | The patient emphasizes the financial strain of medications (e.g., Brilinta) and hospitalizations, including ER and ambulance fees. He highlights how these costs contribute to stress and financial insecurity, directly tying economic factors to his perception of medication intensity as a source of burden. |
| 12 | Concerns over overmedication and side effects | "I think I’m over medicated that way"  "I’ve been bleeding internally … I think that’s caused from stress."  "they put me on … Eliquis … wasn’t very long after that I was right back in here with throwing up blood or pooping blood."  "if you could not remember which medication is for heart? Would that be a problem ever?" | The patient attributes internal bleeding to overmedication with blood thinners (Eliquis), expressing fear of adverse effects. His uncertainty about medication purposes and the physical consequences of treatment highlight his perception of intensity as risky and potentially harmful, amplifying his psychological and physical burden. |
| 12 | Psychological coping through religious faith and acceptance | "I put everything in the Lord’s hands. The Lord’s taking care of everything."  "Isaiah 53:5 says by his stripes we are healed and I just, I’m healed. And I believe in that healing and I’m healed."  "if I go home to be with the Lord, it’s a winning situation also."  "sooner or later everybody’s going to be there anyway." | The patient repeatedly cites religious beliefs as a mechanism to cope with his medical challenges, framing his acceptance of medications and mortality as part of his spiritual faith. This reflects how his psychological resilience shapes his perception of medication intensity as manageable despite the burden. |

**Table S2. Codebook generated by CoTI**

| **Theme name** | **Description** | **Original codes** | **Representative clues** |
| --- | --- | --- | --- |
| Medication Burden | This code captures the emotional and psychological weight associated with managing multiple medications. It reflects patients' feelings of being overwhelmed by the quantity and complexity of their regimens, which can impact adherence and overall quality of life. | "Medication Burden", "Perception of Medication Burden", "Medication Burden and Complexity", "Medication Intensity and Side Effects" | "It’s just a burden to take that many pills.", "I wish I didn't take so much.", "I’m so tired of taking all this medication.", "It’s hard for one person to do it." |
| Financial Burden of Medications | This code encompasses the financial challenges patients face related to their medication costs. It reflects the emotional distress stemming from high prices, insurance issues, and the impact of financial constraints on medication adherence. | "Financial Burden of Medications", "Financial Concerns Related to Medication", "Cost of Medications", "Financial Considerations and Access to Medication" | "I probably spend close to $300 a month on medication.", "You’ve got to make a choice whether you pay your bills or eat.", "It’s been either zero or no copay." |
| Medication Adherence and Management Challenges | This code highlights the difficulties patients encounter in adhering to their medication regimens, including forgetfulness, confusion over dosing, and the need for structured support systems. It emphasizes the importance of strategies to enhance adherence and manage complex regimens. | "Medication Adherence", "Medication Adherence Challenges", "Management of Medication Regimen", "Adherence Challenges" | "I try not to forget. Sometimes I do.", "I just take them. I just, I asked the doctor what’s this for.", "We started doing a pill box a couple days before we came to the hospital this time which has helped." |
| Psychological and Emotional Impact of Medications | This code captures the emotional responses related to medication, including anxiety about adherence, feelings of isolation, and the broader psychological toll that medications can exert on patients' lives. It also encompasses the spiritual beliefs that provide coping mechanisms. | "Psychological Impact", "Medication Adherence Anxiety", "Emotional Impact of Medication", "Social Isolation Due to Medication Effects" | "It was killing my soul.", "I feel so bad. I think about it so much.", "I couldn’t even socialize with my friends." |
| Side Effects and Their Perception | This code relates to the experiences and perceptions of side effects stemming from medications. It highlights how these effects influence patients' willingness to adhere to treatment, their quality of life, and their overall perceptions of medication effectiveness. | "Medication Intensity and Side Effects" "Side Effects Perception" "Concerns about Side Effects of Medications", "Perceived Side Effects of Medications" | "They make me so sleepy.", "I think the Lisinopril would be. The coughing would be considered a side effect.", "I get a little tired of going to the bathroom sometimes." |
| Patient-Doctor Relationship and Communication | This code encompasses patients' perceptions of their interactions with healthcare providers, including concerns about communication gaps and the understanding of treatment plans. It highlights the need for effective patient education and the influence of this relationship on medication adherence. | "Concerns Over Patient Education and Understanding", "Acceptance of Medication Changes with Aging", "Patient Awareness of Heart Failure Diagnosis" | "I don’t think I’ve ever been officially told that.", "Nobody told me.", "I need to do that." |
| Desire for Simplified Medication Regimen | This code reflects patients' preferences for a more streamlined and manageable medication regimen. It captures the sentiment that simpler regimens could enhance adherence and reduce perceived burdens associated with complex dosing schedules. | "Desire for Simplified Medication Regimen", "Desire for Reduced Medication Regimen" | "If she could take less, it would be wonderful.", "I wish I could take them all at once." |
| Perceived Effectiveness of Medications | This code indicates patients' perceptions regarding the effectiveness of their medications and treatment plans. It highlights the emotional complexity tied to treatment efficacy, including feelings of disappointment and hope." | "Perceived Medication Effectiveness", "Perception of Treatment Effectiveness", "Concerns about Medication Efficacy" | "As long as they keep her blood pressure normal.", "I think it’s fine the way it is." |

**Section D**

**Table S3.** Assigned Transcripts for Junior Investigators

| Junior Investigator | Assigned Transcripts |
| --- | --- |
| A | 1, 5, 6, 7, 8, 9, 10 |
| B | 1, 2, 3, 4, 5, 9, 10 |
| C | 1, 2, 3, 7, 8, 9, 10 |
| D | 4, 5, 6, 7, 8, 9, 10 |

**Table S4.** Computation evaluations among three external datasets.

|  | **COVID-19 Dataset** | **Strategy Dataset** |
| --- | --- | --- |
| Clue Similarity (Recall) | 0.732 | 0.675 |
| Theme Similarity (Cosine similarity) | 0.606 | 0.511 |

**Table S5.** Comparison of themes developed by experts and CoTI (without human) on the Sierra Leone Covid-19 interview transcripts.

| **Experts** | **Overlap** | **CoTI** |
| --- | --- | --- |
| Patient experiences  Information | Health workforce  Service delivery  Availability of medicines & supplies  Leadership and governance  Financing  Learning from Ebola | Public Health Communication and Awareness  Patient Access and Public Perception |
|  |  |  |
|  |  |  |
|  |  |  |
|  |  |  |
|  |  |  |

**Table S6.** Comparison of themes developed by experts and CoTI (without human) on the family carers’ strategies interview transcripts.

| **Experts** | **Overlap** | **CoTI** |
| --- | --- | --- |
| Keeping a routine  Providing comfort and reassurance  Activities and interests  Being calm, respectful and avoiding arguments  Allowing the behaviors to continue  Walking away  Time for themselves | Working out and responding to the cause of agitated behaviors  Communication  Keeping people safe  Emotional support  Paid support  Practical support  Understanding agitation as part of dementia | Caregiver strategies for agitation management  Support systems and resources for caregivers  Behavioral changes in dementia patients  Anticipatory grief and loss |
